# History of a Case of Diseased Spleen, Which Terminated Fatally

**Published:** 1817-05

**Authors:** G. D. Yeats

**Affiliations:** King Street, St. James's Square


### HISTORY *of a* CASE *of* DISEASED SPLEEN, *which terminated fatally.*

THE insidious manner in which fatal diseases of the spleen approach, renders a statement of such cases desirable. The unexpectedly sudden termination of the following case, with the appearances on dissection, has induced me to request the insertion of it in your admirably conducted Journal.

Mrs. S——, ætat. 41; married seven months; extremely fat.

Upon visiting this lady, Feb. 28, 1816, the following were the symptoms: She complained of an indescribable general restlessness; some chilliness once in twenty-four hours; sickness, and severe vomiting at times; loss of appetite; thirst; no particular heat of the skin was perceived by the touch, neither did she complain of it, but rather of a sense of coldness; the countenance was, however, flushed at times; the tongue was perfectly dry, and very dark coloured; the bowels were costive; the evacuations, when procured, of a yellow colour, but, by account, they had been very dark; urine high coloured; menses regular; pulse 84, soft; no particular pain was complained of in any part of the body; and on examining the abdomen per tactum, pressure gave no uneasiness in any part of it; her nights were passed in an extremely restless state; there was considerable impatience about her, accompanied by a degree of occasional delirium. At the time of my visit she was much agitated, and underwent a tremor of the muscles approaching to a convulsive paroxysm. She was sometimes faint.—Such is the history of the symptoms which existed at the time of my visit, and which an inquiry into the case procured. Previously to this, she had been for some time subject to nausea and failure of appetite; and, as her husband went to Cheltenham on account of a liver complaint, she availed herself of the opportunity of drinking the waters, which kept her purged, and relieved the sickness. On her return to London, she took calomel twice a week from her physician; but, on the 9th of February, she was seized with distressing sickness, which induced her to take an emetic of ipecac. and antimon. tart. which vomited her very smartly, and brought up much bilious matter. She then (on the 12th) saw her physician again, who ordered her mercurial pills every night, which she took for about three nights, when, in consequence of great nausea and frequent vomiting supervening, her apothecary gave her some nauseating medicines with soda tartarisata. On the 18th, her physician continued these nauseating medicines, but diminished in force; subsequently some other medicines (the cinchona in infusion was one) were taken, which, however, did not agree; nothing indeed seemed to have had any advantage over her complaint. When called to visit her on the 28th of February, the following medicines were directed:

℞. Confect. rosæ ʒvj; aq. cinnamom. simp. f℥vj; aq. distillat. f℥iv. Contere et cola.

℞. Liquoris colati fʒx; acid. sulph. dilut. m. xx; tinct. opii m. iij. M. ft. haust. sextâ quâque horâ sumend.

℞. Olei ricini fʒiij; mucil. acaciæ fʒij, contere addendo tinct. sennæ fʒij; aquæ cinnamom. simp. fʒiv. M. ft. haust. cras mane sumend.

29th. She has passed a quiet night, though without sleep, but she is tranquil to-day. Pulse 78; tongue moist at the edges; bowels moved; evacuations yellow; nausea considerably relieved.

Cont. haust. augendo tinct. opii ad m. iv. Rep. cras mane haust. cathart. addendo tinct. sennæ fʒj.

March 1. Has been much more restless in the night; the other symptoms are the same.

Omit. medicamina.

℞. Submuriat. hydrarg. gr. viij; sacchari pulv. gr. x. M. et divide in chart. ij; sumat. j. hora somni, et alteram post horas vj cum tinct. opii m. vj, et repetatur talis dosis utriusque cras mane.

2d. Seems much better in every respect, but says she does not feel so. Sumat hora somni hydrarg. submuriat. gr. v cum tinct. opii m. xij in pauco aquæ cinnamomi simp.

3d. Had some rest in the night; was tranquil, and felt a return of appetite; the tongue still continues dry. Rep. medicamina et sumat cras mane haustum ex aquæ menthæ virid. f℥jß, magnesiæ sulphat. ℥ß.

4th at noon. Is considerably better in every respect. Pulse 72, soft; tongue moist at the edges, but still dry, and brown in the centre; appetite improved; is in better spirits; bowels moved; evacuations yellow, accompanied with some mucus streaked with blood, and preceded by gripes; gums look a little spongy.

℞. Opii puri pulv. gr. j; hydrarg. submuriat. gr. iij; confect. rosæ q. s. ft. bolus, hoc nocte sumend.

℞. Infusi aurant. comp. fʒx; magnesiæ sulphat. ʒß.

M. ft. haust. statim sumend. et omni mane et meridie repetend.

She passed this day in improved spirits, conversing with some cheerfulness, acknowledging herself better, and desiring that her friends in the country might be immediately informed that she was recovering: ate a boiled egg, and took some ginger beer in the afternoon; slept two hours soundly afterwards, and awoke refreshed. About nine o'clock, while sitting up in bed, she was seized suddenly with sickness and vomiting of yellow bile, accompanied by a fluid evacuation, with some griping; felt extremely faint, and the countenance became exceedingly pale and collapsed, and ghastly; was extremely restless, constantly rising and rolling about the bed, and complaining of want of breath. Pulse not to be felt at the wrist, and she was cold to the touch. After taking some warm brandy and water and an ammonia draught, and rubbing the epigastric region, a considerable quantity of flatus escaped from the stomach, and the pulse returned at the wrist; but still the same restlessness, anxiety, and distressed countenance prevailed, with a constant complaint of tightness in the epigastric region. She heaved for breath; the respiratory muscles laboured exceedingly; the pulse gradually became again obliterated; the restlessness increased, but with more feeble exertions, saying she was dying. A dewy moisture broke out on the forehead; she slipt down in the bed on her back, and calmly expired. She was perfectly collected up to the last moment of her existence. During the progress of her complaint, from the time I first visited her, on the 28th of February, the urine had not deposited any sediment; it remained transparent, and, at times, higher coloured than it ought to be.

The body was opened about twenty hours after death; and the following are the appearances on dissection, with the opinion of the professional gentlemen in attendance, as to the immediate cause of death:

The abdomen was covered with fat three inches in thickness. On cutting into the abdomen, and examining its contents, the stomach was somewhat inflated with air, and its villous coat exhibited signs of considerable morbid vascularity, particularly towards its cardiac orifice. The liver was quite sound in its substance, but its anterior lobe adhered to the diaphragm. The gall-bladder contained very little bile. The spleen was somewhat smaller than natural, and was exceedingly soft; being, in fact, one mass of gore. The lower intestines exhibited much inflammation, with some gangrenous spots. The ovaria were diseased, and contained extravasated blood. Uterus and kidnies sound. No preternatural appearances were observed in the chest, except that the heart was smaller than is usual, and was very flaccid.

"Upon the whole, we are of opinion, that the appearances observed shew sufficient cause of death, and the suddenness of it probably has arisen from a vessel within the spleen giving way, in consequence of its substance being gradually broken down by disease; and we also think, that had life not have been thus cut short, it would not have been prolonged to any distant period."

I confess I was much puzzled to know the precise nature of the disease in this case, until the appearances on dissection detected it. When my opinion was asked, upon being called into consultation, I stated that I could only say what it was not, for that I did not believe it to be a liver complaint; and so far, the appearances on dissection confirmed that opinion. The highly morbid condition of the spleen constituted the most prominent part of the dissection. The extreme quantity of fat, as forming a superfluity of nourishment with which the body was loaded, together with the broken down state of the spleen, naturally suggests the idea that that viscus has no share in the digestive process, as far as the production of nutritious chyle is concerned; otherwise, it is impossible to conceive, that so diseased an organ could, in any way, be connected with such complete nourishment of the body. It appears to me, that this had been a case of diseased spleen from the beginning of the patient's ailments, long before the symptoms assumed a serious aspect; and that when I saw her, the disease was in an incurable state, particularly with a person advanced to the middle period of life with a diseased spleen, as is so accurately described by Dr. Bree, in the second and third volumes of the Medico-Chirurgical Transactions. The vascularity of the villous coat of the stomach might probably have been owing to the efforts of vomiting, determining more blood to that part, or it might not have been altogether morbid, according to the dissections and ideas of Dr. Yelloly, which have thrown much light on this appearance of the stomach. Medico-Chirurgical Transactions, vol. iv.

The appearances on dissection, connected with the history of the symptoms, strike me as supplying matter for comment; and as such, I have troubled you with them.

I am, &c.

G. D. YEATS, M. D.

*King Street, St. James's Square,*
*March* 27, 1817.

